# Regulation of MicroRNAs by Natural Agents: New Strategies in Cancer Therapies

**DOI:** 10.1155/2014/804510

**Published:** 2014-09-01

**Authors:** Neoh Hun Phuah, Noor Hasima Nagoor

**Affiliations:** ^1^Institute of Biological Science (Genetics and Molecular Biology), Faculty of Science, University of Malaya, 50603 Kuala Lumpur, Malaysia; ^2^Centre for Research in Biotechnology for Agriculture (CEBAR), University of Malaya, 50603 Kuala Lumpur, Malaysia

## Abstract

MicroRNAs (miRNAs) are short noncoding RNA which regulate gene expression by messenger RNA (mRNA) degradation or translation repression. The plethora of published reports in recent years demonstrated that they play fundamental roles in many biological processes, such as carcinogenesis, angiogenesis, programmed cell death, cell proliferation, invasion, migration, and differentiation by acting as tumour suppressor or oncogene, and aberrations in their expressions have been linked to onset and progression of various cancers. Furthermore, each miRNA is capable of regulating the expression of many genes, allowing them to simultaneously regulate multiple cellular signalling pathways. Hence, miRNAs have the potential to be used as biomarkers for cancer diagnosis and prognosis as well as therapeutic targets. Recent studies have shown that natural agents such as curcumin, resveratrol, genistein, epigallocatechin-3-gallate, indole-3-carbinol, and 3,3′-diindolylmethane exert their antiproliferative and/or proapoptotic effects through the regulation of one or more miRNAs. Therefore, this review will look at the regulation of miRNAs by natural agents as a means to potentially enhance the efficacy of conventional chemotherapy through combinatorial therapies. It is hoped that this would provide new strategies in cancer therapies to improve overall response and survival outcome in cancer patients.

## 1. Introduction

According to a report by GLOBOCAN, an estimated 14.1 million new cancer cases and 8.2 million cancer deaths were reported, while 32.6 million people are found to be living with cancer (diagnosed in the past five years) in 2012 worldwide. The same report also projected that the number of new cases would increase to 19.3 million by 2025 due to global population aging. In 2012, the cancers of the lung (1.8 million, 13.0% of the total), breast (1.7 million, 11.9%), and colorectum (1.4 million, 9.7%) were the most commonly diagnosed cancers worldwide, while the most common causes of cancer death were cancers of the lung (1.6 million, 19.4% of the total), liver (0.8 million, 9.1%), and stomach (0.7 million, 8.8%) [1].

Cancer is a group of diseases characterized by the uncontrollable growth and spread of abnormal cells which can lead to death without timely intervention. Surgery, radiation, and chemotherapy are among the modalities used in cancer treatment, whose goal is to either cure the disease or prolong and improve the patient's quality of life. Although chemotherapy has led to improvement in this manner, drug resistance and toxicities remain major obstacles to improving the overall response and survival of cancer patients [2]. Drug resistance can be divided into two categories: intrinsic (also known as* de novo*) or acquired resistance [3]. Intrinsic resistance results in ineffective therapy from the start due to presence of resistant phenotype in the tumour cells. On the other hand, acquired resistance develops during treatment whereby tumour cells showed initial responsiveness towards anticancer drugs but attained resistant phenotype during the treatment course. This renders subsequent therapy ineffective leading to tumour recurrence and progression [3]. Hence, there is an urgent need to identify safer but equally effective agents to be used in cancer treatments, which can be found in natural agents.

**Figure 1:**
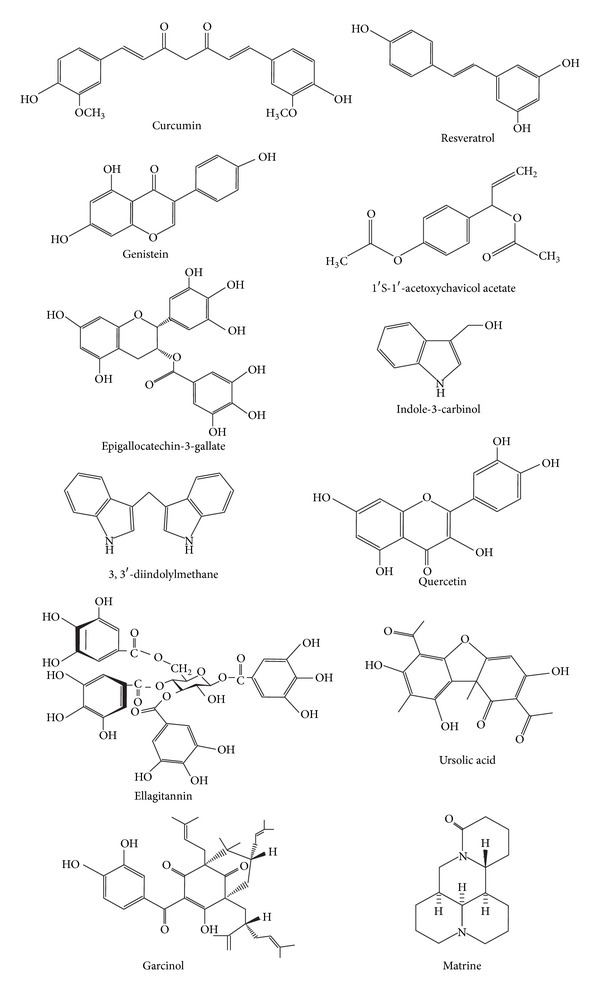
Molecular structures of natural agents regulating miRNAs.

The use of natural agents is promising because not only do they have minimal toxicity to humans compared to conventional chemotherapies, but also they could target numerous signalling pathways. This is beneficial as malignant transformation and progression are multistage processes caused by gene alterations in more than one signalling pathway. This is one of the most plausible explanations why monomodal therapy typically fails in cancer treatments as the specific inhibitors often target only a single gene in a signalling pathway [4]. Therefore, the impact of natural agents on cancer treatment could be more efficacious, as they can be used alone or as adjuvant in combination chemotherapy to improve therapeutic efficacy by overcoming drug resistance and/or reducing drug-induced toxicities. Hence, many of the anticancer agents currently used in cancer therapies have been developed from natural products such as plants (vincristine, vinblastine, etoposide, paclitaxel, camptothecin, topotecan, and irinotecan), marine organisms (cytarabine), and microorganisms (dactinomycin, bleomycin, and doxorubicin) [5]. Besides these, there are also plant-derived dietary polyphenols such as curcumin [6], resveratrol [7, 8], genistein [9], epigallocatechin-3-gallate [10], indole-3-carbinol, and its derivative 3,3′-diindolylmethane [11, 12] (see Figure 1). A number of studies involving cultured cancer cells and animal models have illustrated the protective role of these dietary polyphenols, and mechanistic studies have demonstrated that they exert their antiproliferative and/or proapoptotic effects to prevent the occurrence and/or spread of various cancers by targeting numerous key elements in intracellular signalling network involved in carcinogenesis [13, 14]. Because of the promising results from these* in vitro* and* in vivo* studies, the efficacies of these natural agents in cancer therapies are being investigated in clinical trials (http://www.clinicaltrials.gov/) (see Table 1).

**Figure 2:**
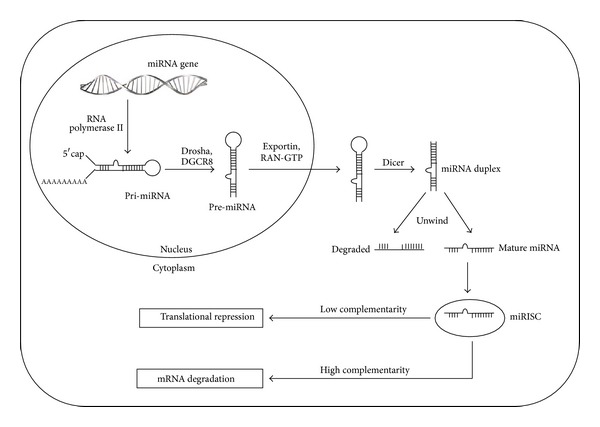
Biogenesis of miRNAs.

MicroRNAs (miRNAs) are highly conserved, small (~22 nucleotides long) noncoding RNA molecules that regulate genes posttranscriptionally [15]. They are predicted to regulate the expression of around 60% of mammalian genes [16] and are found in abundance in many human cell types [17], making them one of the largest class of gene regulators. Although the miRNA genes are mostly found in the intergenic regions, they can also be present in the exonic and intronic regions, in all human chromosomes except for Y chromosome [18]. They are first transcribed by RNA polymerase II as long primary miRNA molecules (pri-miRNAs) before being processed into short hairpin RNAs of ~70 nucleotides known as pre-miRNA by Drosha (RNase III enzyme) and DGCR8 (DiGeorge syndrome critical region 8) [19]. It is then transported into the cytoplasm in a RanGTP-dependent manner by Exportin-5 to be cleaved by another RNase III enzyme, Dicer, to release a ~22 nucleotides long miRNA duplex [20, 21]. One of the strands is incorporated into RNA-induced silencing complex (RISC) which directs miRNA binding to the 3′-untranslated regions (3′ UTR) of target mRNA resulting in mRNA silencing through target cleavage, mRNA degradation, and translation inhibition, while the complementary miRNA strand is usually rapidly degraded [21, 22] (see Figure 2). However, recent studies showed that they can also bind to other regions such as 5′ UTR or coding sequences [23, 24]. Furthermore, they can also upregulate translation of target mRNAs by recruiting proteins complexes to the adenylate-uridylate- (AU-) rich elements of mRNA [25] or interfering with proteins that block the translation of target gene [26]. More interestingly, not only can a single gene be targeted by multiple miRNAs, a single miRNA can also target many genes [27]. miRNAs have been found to be dysregulated in nearly every types of human cancer [28] and various studies have implicated their involvement in a plethora of biological processes, such as tumorigenesis, cell differentiation, proliferation, death, autophagy, metastasis, and drug resistance [29–34]. Hence, identification of these oncogenic or tumour suppressive miRNAs allows for their use as potential targets in cancer therapies [35, 36].

**Figure 3:**
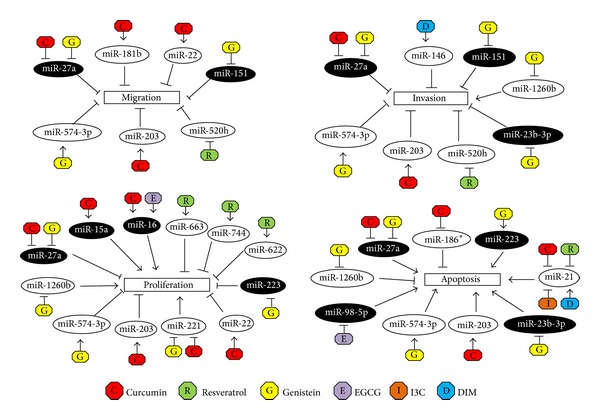
Regulation of miRNAs by natural agents and effects of miRNA inhibition (black ellipse) or overexpression (white ellipse) on cell migration, invasion, proliferation, and apoptosis. Inhibitory relationships are denoted as flat arrow heads, whereas positive interactions are denoted as open arrow heads.

Since natural agents exert their anticancer effects by targeting multiple signalling pathways, and miRNAs regulate diverse biological processes including cell proliferation and programmed cell death, it is thought that miRNAs could play a role in regulating response towards natural agents. Hence, miRNA regulation by natural agents in cancer therapy has been gaining greater attention in recent years. Various studies have reported the dysregulation in the miRNA expression profiles following treatment with natural agents, either as stand-alone or in combination with FDA-approved chemotherapeutic drugs. In one such study, we have previously reported that miRNAs are differentially expressed in response to different treatments, and bioinformatic analysis found their gene targets to be involved in regulating various signalling pathways including apoptosis and cell proliferation [37]. In this review, it was found that most of these dysregulated miRNAs target oncogenes, tumour suppressor genes, and transcription factors (see Tables 2, 3, and 4), and inhibition and/or overexpression of these miRNAs affect cell migration, invasion, proliferation, and apoptosis (see Figure 3). Beside these, there are also studies which found miRNAs to be dysregulated in response to these natural agents, although their targets were not confirmed (these miRNAs are subsequently discussed under “Other miRNAs” section). Therefore, insights from these studies could provide us with a better understanding in the interactions between miRNAs with their specific gene targets and, consequently, help us to delineate the molecular mechanism underlying anticancer drug response.

## 2. Curcumin

Curcumin (diferuloylmethane) is a natural compound derived from the rhizomes of turmeric (*Curcuma longa*) that inhibits cell proliferation, invasion, migration, angiogenesis, and inflammation and induces cell cycle arrest and apoptosis on various cancers, such as breast, cervical, oral, gastric, melanoma, pancreatic, colon, and prostrate [14, 38, 39]. It exhibits its anticancer effects by regulating genes involved in cellular signalling pathways, including nuclear factor-kappa B (NF-*κ*B), protein kinase B (Akt), mitogen-activated protein kinase (MAPK), p53, and other pathways [40].

### 2.1. miRNAs Targeting Oncogenes

Curcumin inhibited proliferation and induced apoptosis through upregulation of miR-181b in MDA-MB-231 breast cancer cells. This miRNA inhibited the expression of matrix metalloproteinases (MMP) by binding to metastases related-cytokines, such as chemokine (C-X-C motif) ligands 1 and 2 (CXCL1 and CXCL2), leading to reduced invasion in both* in vitro* and* in vivo* models. More importantly, it was shown that curcumin upregulated miR-181b and downregulated CXCL1 in tumour cells isolated from primary breast cancers, verifying the clinical significance of their results [41]. Yang et al. reported that curcumin induced apoptosis in MCF-7 breast adenocarcinoma cells through downregulation of B-cell lymphoma 2 (Bcl-2) expression, a key antiapoptotic protein, as well as upregulation of miR-15a and miR-16. Inhibition of these miRNAs restored the expression of Bcl-2 partially and induced cell proliferation [42]. Another study also reported the upregulation of these miRNAs upon treatment in K-562 and HL-60 leukemic cells, and inhibition of these miRNAs partly reversed the downregulation of Wilm's tumour 1 (WT1) induced by curcumin to promote cell growth [43]. A study in 2011 by Saini et al. revealed that curcumin induced hypomethylation of the tumour suppressive miR-203 promoter to increase its expression. This led to downregulation of its two targets, protein kinase B *β* (Akt2) and* v-Src* avian sarcoma viral oncogene homolog (Src), culminating in increased apoptosis as well as decreased proliferation, migration, and invasion in T24 bladder cancer cells [44]. A total of 21 miRNAs were found to be dysregulated (5 upregulated and 16 downregulated) in Y26 retinoblastoma cells treated with curcumin. Among the upregulated miRNAs is miR-22, and its overexpression inhibited cell proliferation and migration. The erythoblastic leukemia viral oncogene homolog 3 (Erbb3) was confirmed as its target in the same study [45].

### 2.2. miRNAs Targeting Tumour Suppressor Genes

Besides that, curcumin suppressed proliferation and induced apoptosis in A549 lung adenocarcinoma cells through downregulation of miR-186∗, suggesting its potential role as an oncogenic miRNA. Interestingly, caspase-10 was identified as a direct target of this miRNA [46]. The same group demonstrated similar effects of miR-186∗ in A549/DDP multidrug-resistant human lung adenocarcinoma cells [47]. In RKO and HCT116 colon cancer cells, the expression of miR-21 which correlated with the inhibition of activator protein-1 (AP-1) binding to its promoter was reduced following treatment with curcumin. Consequently, cell proliferation, tumour growth, invasion, and* in vivo* metastasis were suppressed, while the expression of the tumour suppressor programmed cell death protein 4 (PDCD4), a target of miR-21, was upregulated [48]. In another study, curcumin induced cell cycle arrest and apoptosis through downregulation of Notch-1 specific miR-21 and miR-34a, and upregulation of tumour suppressor let-7a, in TE-7 human esophageal cancer cells [49]. Meanwhile, Liang et al. revealed that curcumin induced apoptosis in HepG2 and HepJ5 hepatocellular carcinoma cells by downregulating Bcl-2 level and upregulating Bcl-2-associated death promoter (BAD) level. Their data indicated that overexpression of miR-200a and miR-200b resulted in resistance towards curcumin through reduced apoptotic effect. A decrease in the level of proapoptotic BAD and Bcl-2-associated X protein (BAX) was found in miR-200a- and miR-200b-over-expressed cells, while an increase in Bcl-2 level was only observed in miR-200b, with no changes in miR-200a-over-expressed cells. Taken together, these data suggest that overexpression of these miRNAs conferred resistance towards curcumin through regulation of the Bcl-2 family [50].

### 2.3. miRNAs Targeting Transcription Factors

Curcumin was found to upregulate 11 and downregulate 18 miRNAs in BxPC-3 human pancreatic adenocarcinoma cells. Of these, the most prominent upregulated miRNA is miR-22 while the most downregulated miRNA is miR-199∗. Importantly, miR-22 upregulation decreased the expression of specificity protein 1 (SP1) and estrogen receptor 1 (ESR1) and vice versa, suggesting that they are targets of miR-22 [78]. The significant relationship between ESR1 gene amplification, which affects the cellular responsiveness to estrogen and antiestrogen, and breast cancer has been reported previously [84, 85]. On the other hand, SP1 proteins play a role in the growth and metastases in various tumour types by regulating cell cycle gene expression and vascular endothelial growth factor receptor [86]. Besides that, curcumin inhibited the growth of RKO and SW480 colon cancer cells through induction of reactive oxygen species (ROS) and repression of specificity proteins (Sp) transcription factors through downregulation of miR-27a, miR-20a, and miR-17-5p. These miRNAs regulate Sp repressors, zinc finger, and BTB domain-containing proteins 4 and 10 (ZBTB4 and ZBTB10) [79]. This has important implications as Sp proteins are transcription factors that regulate genes involved in cell death and angiogenesis and are often overexpressed in tumours [87, 88].

## 3. Resveratrol

Resveratrol (3,4′,5-trihydroxystilbene) is a natural phytoalexin present in several plants, such as grapes, berries, plums, and peanuts. The anticancer effects are mediated through three main mechanisms: inhibition of carcinogenic activation and induction of carcinogen detoxification, induction of growth arrest and apoptosis, and suppression of proinflammatory signalling pathways related to cancer progression [89].

### 3.1. miRNAs Targeting Oncogenes

Treatment with resveratrol in MCF-7 breast adenocarcinoma cells upregulated miR-663 and miR-774, which were able to retard cell proliferation by inhibiting eukaryotic translation elongation factor 1A2 (eEF1A2) at mRNA and protein levels [51]. Liu et al. also reported downregulation of miR-21 by resveratrol in PANC-1, CFPAC-1, and MIA Paca-2 pancreatic cancer cells, leading to inhibition of Bcl-2 expression. Overexpression of miR-21 was found to reverse downregulation of Bcl-2 and resveratrol-induced apoptosis [52]. In anti-benzo[a]pyrene-7,8-diol-9,10-epoxide-transformed human bronchial epithelial cell line (16HBE-T), resveratrol upregulated miR-622, which targets Kirsten rat sarcoma viral oncogene homolog (K-Ras), to inhibit cell proliferation, induce cell cycle arrest at G_0_ phase, and suppress colonies formation* in vitro* and tumorigenicity in nude mice, while its downregulation impaired growth inhibition. Moreover, it was shown that the binding of miR-622 to K-Ras 3′ UTR was affected by resveratrol [53].

### 3.2. miRNAs Targeting Tumour Suppressor Genes

Resveratrol upregulated miR-663 in SW480 human colon cancer cells, besides downregulating various other miRNAs usually found to be overexpressed in this cancer, such as miR-17, miR-21, miR-25, miR-26a, miR-92a-2, miR-103-1 and -103-2, and miR-181a2. The transforming growth factor beta 1 (TGF*β*1) transcript was identified as a target of miR-663 [61]. On the other hand, prostate cancer cells treated with resveratrol displayed downregulation of several miRNAs, including the oncogenic miR-17-92 and miR-106ab clusters. These miRNAs target phosphatase and tensin homologue deleted on chromosome 10 (PTEN), a tumour suppressor protein that is downregulated in nearly all cancers [62]. In another study, resveratrol inhibited cell viability and invasion in highly invasive, androgen independent PC-3 M-MM2 human prostate carcinoma cells, through suppression of Akt. This resulted in the inhibition of miR-21 along with induction of programmed cell death protein 4 (PDCD4) and maspin, targets of miR-21. Similar effects were observed in two other human prostate cancer cells, LNCap and DU145. Additionally, the* in vitro* results were corroborated in severe combined immunodeficient (SCID) mouse xenograft model of prostate cancer, suggesting that resveratrol mediated its effects through inhibition of Akt/miR-21 signalling pathway [63]. Resveratrol also downregulated miR-21 and upregulated PDCD4 in AGS gastric cancer cells. A reduced expression of PDCD4 and overexpression of miR-21 were found in gastric cancer specimens. Interestingly, there was a significant inverse correlation between miR-21 and PDCD4 protein expression, but not between their mRNA expression, suggesting posttranslational modification [64].

### 3.3. miRNAs Targeting Transcription Factors

Besides that, it was found that resveratrol upregulated miR-34a in DLD-1 human colon cancer cells. The E2F transcription factor 3 (E2F3), a target of miR-34a, and E2F3's downstream target sirtuin (silent mating type information regulation 2 homolog) 1 (Sirt1) were downregulated following treatment, suggesting that resveratrol exerted its anticancer activity through miR-34a/E2F3/Sirt1 cascade [80]. In a drug combination study, resveratrol and quercetin induced apoptosis in HT-29 human colon cancer cells by increasing caspase-3 and poly ADP ribose polymerase (PARP) cleavage. The Sp1, Sp3, and Sp4 specificity proteins, together with Sp-regulated antiapoptotic protein, survivin, were reduced at both mRNA and protein levels. Moreover, this combination treatment also decreased miR-27a and induced ZBTB10 [90]. In a study involving CLI-5 and A549 lung adenocarcinoma cells, resveratrol downregulated miR-520h and triggered miR-520h-mediated signal cascade, resulting in inhibition of forkhead box C2 (FOXC2) and subsequent suppression of tumour metastasis in both* in vitro* and* in vivo* models. The same study showed association between expression of FOXC2 with epithelial-mesenchymal transition (EMT), cell motility, metastasis, and poorer prognosis in lung cancer patients [81]. Resveratrol upregulated miR-663 in THP-1 human monocytic cells and human blood monocytes. MiR-663 decreased the activity of AP-1 by targeting JunB and JunD transcripts, and its upregulation decreased the levels of miR-155, which has been linked to formation and development of tumours such as breast, gastric, and lung cancers [82, 91].

### 3.4. Other miRNAs

In A549 human lung adenocarcinoma cells, resveratrol altered miRNA expression profiles, with downregulated miR-92a-2∗ and upregulated miR-299-5p, miR-194∗, miR-338-3p, miR-758, and miR-582-3p exhibiting greater than 20-fold changes. Importantly, identification of their targets through bioinformatics analyses revealed their involvement in regulating apoptosis, cell cycle, cell proliferation, and cell differentiation [92]. In another study, a number of tumour suppressive miRNAs including miR-141 and miR-200c were upregulated by resveratrol, inhibiting the breast cancer stem-like cell characteristics in MDA-MB-231-luc-D3H2LN cells (a luciferase expressing cell line that was derived from MDA-MB-231 human breast adenocarcinoma cells). Repression of miR-141 resulted in increased cell invasion [93]. In p53 wild-type (MCF-7) and p53 mutant-type (MDA-MB-231 and BT-549) breast cancer cells, resveratrol suppressed pAkt and phosphorylation of CCAAT/enhancer binding protein beta (C/EBP-*β*), which has been identified as a negative regulator of miR-145, leading to upregulation in this miRNA [94]. More recently, it was reported that resveratrol downregulated the expression of DNA methyltransferase 3b (DNMT3b) and upregulated miR-21, miR-129, miR-204, and miR-489 in hormone sensitive mammary tumours, but vice versa in matched normal mammary tissue. An inverse association between DNMT3b and miR-129, miR-204, and miR-489 expression in normal and tumour tissue was also found [95].

## 4. Genistein

Genistein is a isoflavone naturally found in numerous plants, including fava beans, lupins, and soybeans [96]. Previous reports have shown that it induces cell cycle arrest and apoptosis and inhibits angiogenesis and metastasis in various cancers including breast, prostate, gastric, lung, pancreatic, melanoma, and renal cancer in both* in vitro* and* in vivo* models. Besides having antioxidant properties, it also inhibits NF-*κ*B and Akt signalling pathways and antagonizes estrogen- and androgen-mediated signalling pathways [97].

### 4.1. miRNAs Targeting Oncogenes

In PC-3 and DU145 prostate cancer cells, genistein upregulated miR-34a with HOX transcript antisense RNA (HOTAIR) confirmed as its target. Knockdown of this gene decreased cell proliferation, migration, and invasion, and induced apoptosis and cell cycle arrest [54]. Also in the same cells, genistein upregulated miR-574-3p and overexpression of this miRNA inhibited cell proliferation, migration, and invasion* in vitro* and* in vivo*, and apoptosis was induced through reduction of B cell lymphocyte xL (Bcl-xL) and activation of caspase-3 and -9. The Ras-related C3 botulinum toxin substrate 1 (RAC1), epidermal growth factor receptor (EGFR), and E1A binding protein p300 (EP300) were confirmed as targets of miR-574-3p, and inhibition in cell proliferation, migration, and invasion was observed when these three targets were knocked down. The expression of miR-574-3p was found to be significantly lower in PC-3 and DU145 and prostate tumour tissues compared with RWPE-1 normal prostate cells and adjacent normal tissues, respectively. The low expression level of miR-574-3p correlated with advanced tumour stage and higher Gleason score [55]. Also in PC-3 prostate cancer cells, genistein upregulated miR-1296 and suppressed the expression of minichromosome maintenance (MCM) gene family, which is essential for DNA replication and frequently found to be upregulated in various cancers. Inhibition of miR-1296 resulted in upregulation of MCM2 mRNA and protein and vice versa, strongly suggesting MCM2 as its target. Furthermore, MCM2 expression correlated with prostate cancer progression, whereby reduced expression of miR-1296 was observed in prostate carcinoma compared to benign prostate hyperplasia [56].

### 4.2. miRNAs Targeting Tumour Suppressor Genes

Genistein also downregulated miR-221 and miR-222 in PC-3 prostate cancer cells, leading to upregulation of aplysia ras homolog I (ARH1), a target of these miRNAs. Overexpression of ARH1 led to inhibition of cell proliferation, colony formation, and invasion [65]. It was reported that miR-151 has higher expression in PC-3 and DU145 prostate cancer cells compared to RWPE-1, a nonmalignant epithelial prostate cell line. Genistein downregulated miR-151 and its suppression inhibited cell migration and invasion but not cell proliferation in the prostate cancer cells. Moreover, miR-151 was also shown to bind directly to the 3′ UTRs of NEDD4 binding protein 1 (N4BP1), castor zinc finger 1 (CASZ1), interleukin 1 receptor accessory protein-like 1 (IL1RAPL1), SRY (sex determining region Y)-box 17 (SOX17), and Rho GDP dissociation inhibitor (GDI) alpha (ARHGDIA). It was found that miR-151 expression is significantly higher in prostate cancer compared to the benign state, although a higher expression did not show significant correlation with lower survival rate [66]. Another miRNA whose expression is inhibited by genistein is miR-23b-3p, and its knockdown in A-498 and Caki-2 renal cancer cells resulted in significant apoptosis induction and reduction in invasive capabilities, but not cell cycle progression. Furthermore, miR-23b-3p was found to directly target PTEN and its inhibition induced PTEN expression with concomitant reduction in PI3-kinase, total Akt, and IL-32. The expression level of this miRNA was found to be inversely correlated with five-year survival rate in renal cancer patients, and lack of PTEN protein expression was observed in tissue samples with high miR-23b-3p expression [67]. Also in renal cancer cells, genistein significantly decreased the expression of miR-1260b in 786-O and A-498 cells and TCF reporter activity was found to be paralleled to its expression, indicative of its regulation of the *β*-catenin-dependent pathway. Furthermore, miR-1260b promoted cell proliferation and invasion while apoptosis was reduced in these cells. Tumour suppressors associated with Wnt-signalling such as secreted frizzled-related protein 1 (sFRP1), dickkopf 2 homolog (Dkk2), and mothers against decapentaplegic 4 (Smad4) were confirmed as targets of miR-1260b, and their overexpression decreased cell proliferation and invasion along with increased apoptosis. More pertinently, miR-1260b has higher expression in renal cancer tissues compared to normal kidney tissues and its expression significantly correlates with shorter overall survival [68]. Similar results with miR-1260b were reported in PC-3 and DU-145 prostate cancer cells, suggesting the importance and potential therapeutic benefit of this miRNA in cancer treatment [69]. In a study by Ma et al., it was demonstrated that genistein downregulated miR-223 and upregulated its targets, F-box and WD-40 domain protein 7 (Fbw7). In addition, inhibition of miR-223 retarded cell growth and induced apoptosis in pancreatic cancer cells [70]. Genistein inhibited the expression of miR-27a in C918 uveal melanoma [71], SKOV3 ovarian cancer [72], and pancreatic cancer cells [73]. The expression of ZBTB10 and Sprouty2, targets of miR-27a, was found to be upregulated following treatment, while inhibition of miR-27a suppressed cell growth, migration, and invasion and induced apoptosis in these cells [71–73]. In PC-3 prostate cancer cells, genistein in combination with 5-aza-20-deoxycytidine (5-aza) and trichostatin A (TSA) upregulated miR-145. Loss of miR-145 expression was frequently observed in prostate cancer cell lines and tissue samples compared to normal cells and matched adjacent normal tissues, respectively. This inactivation was seen as a result of DNA methylation in the miR-145 promoter region. Overexpression of miR-145 resulted in decreased cell viability, increased apoptosis, cell cycle arrest, and upregulation of proapoptotic tumour necrosis factor (ligand) superfamily, member 10 (TNFSF10) [98].

### 4.3. Other miRNAs

Various miRNAs were found to be dysregulated following treatment with genistein in prostate cancer cells: one upregulated (miR548b-3p) and four downregulated miRNAs (miR-15b, miR-125a, miR-125b, and miR-320) in PC-3 cells; five downregulated miRNAs (miR-155, miR-208b, miR-211, miR-376a, and miR-411) in DU-145 cells; one upregulated miRNA (miR-15a), and three downregulated miRNAs (miR-494, miR-520g, and miR-542) in LNCap cells. These results showed that miRNAs are differentially expressed in different prostate cancer cells treated with genistein, although their roles were not investigated in the same study [99]. Genistein also inhibited miR-21 in A-498 renal cancer cells and tumour xenografts, leading to cell cycle arrest, apoptosis induction, reduced invasion, and migration capabilities as well as increased expression of p21 and p38 MAPK but a reduction in cyclin E2. This study showed that patients with lower miR-21 expression have better five-year survival rate while increased expression correlates with an increased stage of renal cancer, providing clinical relevance in the role of miR-21 in renal cancer [100]. Additionally, genistein upregulated miR-34a in pancreatic cancer cells. Treatment with genistein and overexpression of miR-34a inhibited cell growth and induced apoptosis with concomitant downregulation of Notch-1 signalling pathway [101].

## 5. Epigallocatechin-3-Gallate (EGCG)

The epigallocatechin-3-gallate (EGCG) is the major polyphenol found in green tea (*Camellia sinensis*) with anticancer effects. Previous reports have shown that it is able to suppress proliferation, induce apoptosis, and inhibit invasion, angiogenesis, and metastasis in various cancer types in both* in vitro* and* in vivo* models by targeting multiple cellular signalling pathways [102, 103].

### 5.1. miRNAs Targeting Oncogenes

Treatment with EGCG in HepG2 hepatocellular carcinoma cells altered the expression of 61 miRNAs (13 upregulated and 48 downregulated) and among the upregulated miRNAs is miR-16. Its target, Bcl-2, was also downregulated by EGCG. Suppression of miR-16 counteracted the effects of EGCG on apoptosis induction and Bcl-2 downregulation [57].

### 5.2. miRNAs Targeting Tumour Suppressor Genes

Besides that, it was also reported that EGCG enhanced the effects of cisplatin by downregulating miR-98-5p in A549 lung cancer cells. Inhibition of miR-98-5p enhanced cisplatin-induced cell death and increased expression of p53 [74].

### 5.3. Other miRNAs

In HepG2 hepatocellular carcinoma cells, five miRNAs (miR-30b∗, miR-453, miR-520-e, miR-629, and miR-608) were downregulated following treatment with EGCG. Bioinformatics analysis revealed gene targets of miR-30b∗ to be involved in regulating various pathways including inflammation, NF-*κ*B, peroxisome proliferator-activated receptor (PPAR) signalling, insulin signalling, glycolysis and gluconeogenesis, glycerolipid metabolism, mitochondria and oxidative phosphorylation, and glutathione metabolism [104]. EGCG upregulated miR-210, a key component of hypoxia-inducible factor 1α (HIF-1*α*), through the hypoxia-response element found in the promoter region, in both H1299 and H460 non-small-cell lung cancer and CL13 mouse lung adenocarcinoma cell lines. Overexpression of miR-210 reduced cell proliferation rate and anchorage-independent growth [105]. In a study by Chakrabarti et al., it was reported that EGCG downregulated three oncogenic miRNAs (miR-92, miR-93, and miR-106b) and upregulated three tumour suppressive miRNAs (miR-7-1, miR-34a, andmiR-99a), leading to induction of both intrinsic and extrinsic apoptotic pathways in SK-N-BE2 and IMR-32 malignant neuroblastoma cell lines. The alterations in expression of these miRNAs were found to be more significant when treated with EGCG in combination with N-(4-hydroxyphenyl) retinamide (4-HPR), and overexpression of miR-93 and miR-7-1, which exhibited the highest fold-change, decreased and increased efficacy towards EGCG, respectively [106]. The 4-HPR is a synthetic retinoid that has been reported by Reynolds et al., to induce differentiation of neuroblastoma cells and increase survival rates in neuroblastoma patients [107]. Overexpression of miR-93 and miR-7-1, which exhibited the highest fold-change, decreased and increased efficacy towards EGCG, respectively [107]. Similar results were reported on SH-SY5Y and SK-N-DZ malignant neuroblastoma cell lines when used as stand-alone agent [108]. Meanwhile, it was found that oncogenic miR-21 was downregulated while tumour suppressive miR-330 was upregulated in prostate cancer xenograft tissues of EGCG-treated mice [109].

## 6. Indole-3-Carbinol (I3C) and 3,3′-Diindolylmethane (DIM)

The indole-3-carbinol (I3C) is a naturally occurring glucosinolates found in the* Brassica* vegetables such as cabbage, broccoli, cauliflower, kale, radish, turnip, and brussels sprouts, while 3,3′-diindolylmethane (DIM) is a prominent product obtained when I3C undergoes condensation reactions in the stomach [110]. Both I3C and DIM have been reported to modulate many genes involved in regulating cell cycle, cell proliferation, signal transduction, apoptosis, and other cellular processes [111, 112].

### 6.1. miRNAs Targeting Oncogenes

Treatment with DIM upregulated mR-21 and downregulated its target, cell division cycle 25 homolog A (Cdc25A), leading to reduced cell proliferation in MCF-7 breast cancer cells. Consequently, repression of miR-21 elevated Cdc25A level, while treatment with DIM partially restored Cdc25A expression and enhanced cell proliferation [58]. It was also reported that DIM upregulated the expression of the let-7 family and reduced the expression of its target, histone-lysine N-methyltransferase (EZH2), to inhibit self-renewal and clonogenic capacity in LNCaP, C4-2B, and PC-3 prostate cancer cell lines. The loss of let-7 family was also found to be inversely correlated with increased expression of EZH2 in prostate cancer tissues compared to adjacent normal prostate tissues [59]. The expression of miR-146a was found to be lower in pancreatic cancer cells compared with normal human pancreatic duct epithelial cells. Treatment with DIM (which increased miR-146a expression) or reexpression of miR-146a downregulated EGFR, interleukin 1 receptor-associated kinase 1 (IRAK-1), NF-*κ*B and metastasis-associated protein, member 2 (MTA2), blocking cell invasion in Colo357 and Panc-1 pancreatic cancer cells [60].

### 6.2. miRNAs Targeting Tumour Suppressor Genes

The upregulation of miR-21, miR-31, miR-130a, miR-146b, and miR-377 observed in vinyl carbamate-induced lung cancer in mice was reversed by I3C. In addition, PTEN, PDCD4, and reversion-inducing-cysteine-rich protein with Kazal motifs (RECK) were identified as potential targets of miR-21 [75]. In another study, it was demonstrated that I3C also downregulated miR-21 in Panc-1 pancreatic carcinoma cells. Overexpression of miR-21 negated I3C-induced sensitivity towards gemcitabine and reduced the expression of its target, PDCD4, which was upregulated by I3C [76]. DIM was also found to downregulate miR-221 and upregulates its targets: PTEN, cyclin-dependent kinase inhibitor 1B (p27^kip1^), cyclin-dependent kinase inhibitor 1C (p57^kip2^), and p53 up-regulated modulator of apoptosis (PUMA), leading to suppression of cell proliferation and migration of MiaPaCa-2 and Panc-1 pancreatic cancer cells. MiR-221 was significantly upregulated in pancreatic cancer cell lines and tumour tissues compared to normal pancreatic duct epithelial cells and tissues. Furthermore, patients with high expression of miR-221 had a relatively shorter survival compared to those with lower expression [77].

### 6.3. miRNAs Targeting Transcription Factors

The expression of miR-200b, miR-200c, let-7b, let-7c, let-7d, and let-7e was found to be downregulated in gemcitabine-resistant MiaPaCa-2 pancreatic cancer cells following treatment with DIM. The zinc finger E-box binding homeobox 1 (ZEB1), target of miR-200, was found to be downregulated following reexpression of miR-200. In addition, slug and vimentin were also downregulated, while E-cadherin was upregulated. These changes were consistent with phenotypic reversal of mesenchymal to epithelial morphology [83]. Besides that, DIM enhanced the effects of Herceptin when used in combination by inhibiting cell growth, colony formation, and inducing apoptosis in SKBR3 and MDA-MB-468 breast cancer cells through upregulation of miR-200. This combination treatment and overexpression of miR-200 resulted in downregulation of FoxM1, which has been implicated in progression of various cancers [113].

### 6.4. Other miRNAs

In another study, DIM downregulated miR-92a which is known to be associated with receptor activator of nuclear factor-*κ*B ligand (RANKL) signalling, EMT, and cancer progression to inhibit differentiation of osteoclasts and osteoblasts in prostate cancer metastasis [114].

## 7. Other Natural Agents

Ellagitannin (1,3-Di-O-galloyl-4,6-(s)-HHDP-b-D-glucopyranose), a polyphenolic compound isolated from* Balanophora japonica*, was reported to upregulate 17 miRNAs and downregulate 8 miRNAs in HepG2 hepatocellular carcinoma cells [115]. Another study reported that ursolic acid, a pentacyclic triterpene acid found in medicinal herbs such as* Oldenlandia diffusa* and* Radix actinidiae*, induced apoptosis in U251 glioblastoma cells by downregulating miR-21 and inducing the expression of PDCD4, a target of miR-21. Moreover, it was shown that overexpression of miR-21 suppressed the ursolic acid-induced expression of PDCD4 [116]. Garcinol, a polyisoprenylated benzophenone derivative obtained from* Garcinia indica* extracts, was found to reverse EMT in MDA-MB-231 and BT-549 breast cancer cells as well as in xenograft mouse model through upregulation of let-7a, let-7e, let-7f, miR-200b, and miR-200c. Additionally, it was also demonstrated that inhibition of miR-200a, miR-200b, and miR-200c attenuated the garcinol-mediated inhibition of invasion [117]. Besides that, matrine, an alkaloid isolated from* Sophora flavescens*, downregulated miR-21 to induce overexpression of PTEN and inactivate Akt, leading to cell cycle arrest and apoptosis in MCF-7 breast cancer cells [118]. Quercetin, a flavonoid found in onions, apples, tea, and red wine, upregulated miR-142-3p in pancreatic ductal adenocarcinoma cells (MIA PaCa-2, Capan-1, and S2-013). Overexpression of miR-142-3p inhibited cell proliferation and reduced the expression of its target, heat-shock protein 70 (HSP7). It was also shown that miR-143-3p regulated HSP70 expression independent of HSP1 and overexpression of HSP70 rescued miR-143-3p-induced cell death [119]. Furthermore, upregulation of miR-146a, a negative regulator of NF-*κ*B activation, by quercetin protected CCD-180Co colonic myofibroblast cells against ROS [120]. Recently, we have also reported that a total of 25 miRNAs were found to be significantly differentially expressed following treatment with 1′S-1′-acetoxychavicol acetate (ACA), a natural compound isolated from the wild ginger,* Alpinia conchigera*, and/or cisplatin on Ca Ski and HeLa cervical carcinoma cells. These include miR-138, miR-210, and miR-744 with their predicted targets involved in signalling pathways regulating apoptosis and cell cycle progression [37]. Another study reported that ACA also downregulated miR-23a in HN4 head and neck squamous cells and its inhibition suppressed cell proliferation and induced apoptosis, with PTEN confirmed as its target [121]. Sulforaphane, an isothiocyanate derived from cruciferous vegetables such as broccoli and broccoli sprouts, upregulated 15 miRNAs (miR-372, miR-342-3p, miR-486-5p, miR-9, miR-9∗, miR-145, miR-146a, miR-629, miR-505, miR-758, miR-30a∗, miR-27b∗, miR-135b∗, miR-27b, and miR-23b) and downregulated 3 miRNAs (miR-633, miR-155, and miR-106a∗) in NCM460 and NCM356 normal colon epithelial cells [122]. In sulforaphane-treated T24 bladder cancer cells, miR-200c was found to be upregulated, leading to inhibition of EMT and metastasis. Downregulation and overexpression of miR-200c reversed and enhanced ZEB1 repression and E-cadherin induction by sulforaphane, respectively [123]. Besides that, miR-140 was found to be upregulated following sulforaphane treatment in MCF10DCIS and MDA-MB-231 breast cancer cells. Both SOX9 and aldehyde dehydrogenase 1 (ALDH1) were also identified as targets of miR-140, and miR-140 overexpression downregulated the protein levels in both targets [124].

## 8. Conclusion

Based on current literature, it is increasingly evident that natural agents such as curcumin, resveratrol, genistein, EGCG, I3C, and DIM can alter miRNA expression profiles and target multiple genes at the same time. The pleiotropic effects of natural agents and miRNAs are attractive for cancer therapy since cancer is caused by defects in multiple genes. For this reason, it is proposed that natural agents can be used to downregulate oncogenic miRNAs and upregulate tumour suppressive miRNAs to restore drug sensitivity. Hence, there is a more promising role for natural agents in cancer therapies beyond their current use as chemopreventive agents and supplements. In conclusion, these natural agents which have been tested extensively in clinical trials and found to possess encouraging safety profiles can be used to simultaneously target diverse cellular signalling via specific miRNAs to serve as potential new avenues in cancer treatments. Together, it is hoped that these new strategies would prevent tumour recurrence and resistance towards conventional therapies, leading to improvement in the overall response and survival of cancer patients.

## Figures and Tables

**Table 1:** Clinical trials involving selected natural agents.

Natural agent	Trial identifier	Phase	Cancer type	Status
Curcumin	NCT01042938	2	Breast	Completed
	NCT00094445	2	Pancreatic	Completed
	NCT00192842	2	Pancreatic	Completed
	NCT01333917	1	Colorectal	Completed
	NCT00027495	1	Colorectal	Completed
	NCT01035580	1	Uterine cervical	Completed
	NCT00113841	n.a	Multiple myeloma	Completed
	NCT01975363	n.a	Breast	Active
	NCT01859858	1	Colorectal	Active
	NCT00745134	2	Rectal	Active
	NCT01294072	1	Colon	Active
	NCT01917890	n.a	Prostate	Active
	NCT01160302	0	Head and neck	Active
	NCT01490996	1/2	Colon	Active
	NCT02095717	2	Prostate	Active
	NCT02017353	2	Endometrial	Active
	NCT02064673	2	Prostate	Active
	NCT00641147	n.a	Familial adenomatous Polyposis	Active
	NCT00927485	n.a	Familial adenomatous Polyposis	Active
	NCT01948661	2	Colorectal	Active

Resveratrol	NCT00256334	1	Colon	Completed
	NCT00433576	1	Colorectal	Completed
	NCT00098969	1	Solid tumours	Completed
	NCT01476592	n.a	Neuroendocrine	Active

Genistein	NCT00244933	2	Breast	Completed
	NCT00118040	2	Bladder	Completed
	NCT00099008	1	Breast	Completed
	NCT00584532	2/3	Prostate	Completed
	NCT00376948	2	Pancreatic	Completed
	NCT00269555	n.a	Prostate	Completed
	NCT00499408	2	Prostate	Completed
	NCT00078923	2	Prostate	Completed
	NCT01985763	1/2	Colorectal	Active
	NCT01325311	2	Prostate	Active
	NCT01126879	2	Prostate	Active
	NCT01628471	1/2	Lung	Active
	NCT00276835	0	Kidney; melanoma	Active
	NCT01182246	1/2	Pancreatic	Active

EGCG	NCT00459407	1	Prostate	Completed
	NCT00233935	1	Esophageal	Completed
	NCT00573885	2	Lung	Completed
	NCT00303823	2	Cervical	Completed
	NCT01105338	2/3	Prostate	Completed
	NCT00516243	1	Breast	Active
	NCT00942422	2	Multiple myeloma	Active
	NCT00596011	2	Prostate	Active
	NCT01606124	2	Colorectal	Active
	NCT00253643	n.a	Prostate	Active
	NCT00917735	2	Breast	Active
	NCT01360320	2	Colorectal	Active
	NCT00949923	1	Breast	Active

I3C	NCT00607932	n.a	Prostate	Completed
	NCT00033345	1	Breast	Completed
	NCT00100958	1	Solid tumours	Completed

DIM	NCT00450229	1	Prostate	Completed
	NCT00305747	1	Prostate	Completed
	NCT00462813	3	Cervical	Completed
	NCT00888654	2	Prostate	Active
	NCT01391689	2/3	Breast	Active

**Table 2:** Selected miRNAs targeting oncogenes.

Natural agent	miRNA	Regulation	Target(s) and function(s)	Cancer type	Reference
Curcumin	miR-181b	Up	Targets CXCL1 and CXCL2; inhibited migration through MMP	Breast	[41]
	miR-15a, miR-16	Up	miRNA inhibition upregulates Bcl-2 and WT1 and induces cell growth	Breast; leukemia	[42, 43]
	miR-203	Up	Targets Akt2 and Src; inhibits cell proliferation, invasion, and migration; and induces cell cycle arrest and apoptosis	Bladder	[44]
	miR-22	Up	Targets Erbb3; inhibits cell proliferation and migration	Pancreatic	[45]

Resveratrol	miR-663, miR-744	Up	Targets eEF1A; inhibits cell proliferation	Breast	[51]
	miR-21	Down	Upregulates Bcl-2; increases apoptosis	Pancreatic	[52]
	miR-622	Up	Targets K-Ras; inhibits proliferation and colony formation *in vitro* and tumorigenicity *in vivo *	Lung	[53]

Genistein	miR-34a	Up	Targets HOTAIR	Prostate	[54]
	miR-574-3p	Up	Targets RAC1, EGFR, and EP300; inhibits cell proliferation, migration, and invasion; and induces apoptosis	Prostate	[55]
	miR-1296	Up	miRNA inhibition upregulates MCM2 and vice versa	Prostate	[56]

EGCG	miR-16	Up	Targets Bcl-2	Hepatocellular	[57]

DIM	miR-21	Up	Targets Cdc25A	Pancreatic	[58]
	let-7 family	Up	Downregulates EZH2	Prostate	[59]
	miR-146	Up	Downregulates EGFR, IRAK-1, NF-kB, and MTA2; inhibits cell invasion	Pancreatic	[60]

**Table 3:** Selected miRNAs targeting tumour suppressor genes.

Natural agent	miRNA	Regulation	Target(s) and function(s)	Cancer type	Reference
Curcumin	miR-186∗	Down	Targets caspase-10; inhibits apoptosis	Lung	[46, 47]
	miR-21	Down	Targets PDCD4	Colon	[48]

Resveratrol	miR-663	Up	Targets TGF*β*1	Colon	[61]
	miR-17-92, miR-10ab	Down	Targets PTEN	Prostate	[62]
	miR-21	Down	Targets PDCD4 and maspin	Prostate	[63, 64]

Genistein	miR-221, miR-222	Down	Targets ARH1	Prostate	[65]
	miR-151	Down	miRNA inhibition suppresses cell migration and invasion; targets N4BP1, CASZ1, IL1RAPL1, SOX17, and ARHGDIA	Prostate	[66]
	miR-23b-3p	Down	Targets PTEN; miRNA inhibition induces apoptosis and inhibits invasion	Renal	[67]
	miR-1260b	Down	Targets sFRP1, Dkk2 and, Smad4; induces cell proliferation and invasion; inhibits apoptosis	Renal; Prostate	[68, 69]
	miR-223	Down	Upregulates Fbw7; miRNA inhibition suppresses cell growth and induces apoptosis	Pancreatic	[70]
	miR-27a	Down	Upregulates ZBTB10 and Sprouty2; miRNA inhibition suppresses cell growth, migration, and invasion and induces apoptosis	Melanoma; Ovarian; Pancreatic	[71–73]

EGCG	miR-98-5p	Down	miRNA inhibition enhances cisplatin-induced apoptosis and increases p53 expression	Lung	[74]

I3C	miR-21	Down	Downregulates PTEN, PDCD4, and RECK	Lung; Pancreatic	[75, 76]
	miR-221	Down	Upregulates PTEN, p27kip1, p57kip2, and PUMA; increases cell proliferation	Pancreatic	[77]

**Table 4:** Selected miRNAs targeting transcription factors.

Natural agent	miRNA	Regulation	Target(s) and function(s)	Cancer type	Reference
Curcumin	miR-22	Up	miRNA inhibition upregulates SP1 and ESR1 expression and vice versa	Pancreatic	[78]
	miR-27a, miR-20a, miR-17-5p	Down	Targets ZBTB4 and ZBTB10	Colon	[79]

Resveratrol	miR-34a	Up	Targets E2F3	Colon	[80]
	miR-520h	Down	Inhibits cell migration and invasion, decreases levels of FOXC2	Lung	[81]
	miR-663	Up	Targets JunB and JunD; decreases miR-155	Leukemia	[82]

DIM	miR-200	Down	Targets ZEB1; downregulates ZEB1, slug, and vimentin; upregulates E-cadherin	Pancreatic	[83]

## Abbreviations

Ago2: Argonaute2
Akt: Protein kinase B
Akt2: Protein kinase B *β*
ALDH1: Aldehyde dehydrogenase 1
AP-1: Activator protein-1
ARH1: Aplysia ras homolog 1
ARHGDIA: Rho GDP dissociation inhibitor (GDI) alpha
BAD: Bcl-2-associated death promoter
BAX: Bcl-2-associated X protein
Bcl-2: B-cell lymphoma 2
Bcl-xL: B-cell lymphocyte xL
CASZ1: Castor zinc finger 1
Cdc25A: Cell division cycle 25 homolog A
C/EBP-*β*: CCAAT/enhancer binding protein beta
CXCL: Chemokine (C-X-C motif)
DNMT3b: DNA methyltransferase 3b
Dkk2: Dickkopf 2 homolog
eEF1A: Eukaryotic translation elongation factor 1A
EGFR: Epidermal growth factor receptor
EMT: Epithelial-mesenchymal transition
EP300: E1A binding protein p300
Erbb3: Erythoblastic leukemia viral oncogene homolog 3
ESR1: Estrogen receptor 1
Fbw7: F-box and WD-40 domain protein 7
FOXC2: Forkhead box C2
HIF-1α: Hypoxia-inducible factor 1*α*
HOTAIR: HOX transcript antisense RNA
HSP70: Heat-shock protein 70
IL1RAPL1: Interleukin 1 receptor accessory protein-like 1
IRAK-1: Interleukin 1 receptor-associated kinase 1
K-Ras: Kirsten rat sarcoma viral oncogene homolog
MAPK: Mitogen-activated protein kinase
MCM: Minichromosome maintenance
MMP: Matrix metalloproteinases
MTA2: Metastasis-associated protein, member 2
N4BP1: NEDD4 binding protein 1
NF-*κ*B: Nuclear factor-kappa B
p27^kip1^: Cyclin-dependent kinase inhibitor 1B
p57^kip2^: Cyclin-dependent kinase inhibitor 1C
PARP: Poly ADP ribose polymerase
PDCD4: Programmed cell death protein 4
PPAR: Peroxisome proliferator-activated receptor
PTEN: Phosphatase and tensin homologue deleted on chromosome 10
PSA: Prostate specific antigen
PUMA: p53 upregulated modulator of apoptosis
RAC1: Ras-related C3 botulinum toxin substrate 1
RANKL: Receptor activator of nuclear factor-*κ*B ligand
RECK: Reversion-inducing-cysteine-rich protein with Kazal motifs
ROS: Reactive oxygen species
sFRP1: Wnt-signalling secreted frizzled-related protein 1
Sirt1: Sirtuin (silent mating type information regulation 2 homolog) 1
SMAD: Mothers against decapentaplegic
Sp: Specificity protein
Src: V-src avian sarcoma viral oncogene homolog
SOX17: SRY (sex determining region Y)-box 17
TGF*β*: Transforming growth factor beta
TNFSF10: Tumour necrosis factor (ligand) superfamily, member 10
WT1: Wilm's tumour 1
ZBTB: Zinc finger and BTB domain-containing protein
ZEB1: Zinc finger E-box binding homeobox 1.
